# Wounded but unstressed: Moose tolerate injurious flies in the boreal forest

**DOI:** 10.1093/jmammal/gyae081

**Published:** 2024-08-07

**Authors:** Bridgett M Benedict, Daniel P Thompson, John A Crouse, Gabriel L Hamer, Perry S Barboza

**Affiliations:** Department of Ecology and Conservation Biology, Texas A&M University, College Station, TX 77843, United States; Alaska Department of Fish and Game, Kenai Moose Research Center, Soldotna, AK 99669, United States; Alaska Department of Fish and Game, Kenai Moose Research Center, Soldotna, AK 99669, United States; Department of Entomology, Texas A&M University, College Station, TX 77843, United States; Department of Ecology and Conservation Biology, Texas A&M University, College Station, TX 77843, United States; Department of Rangelands Wildlife and Fisheries Management, Texas A&M University, College Station, TX 77843, United States

**Keywords:** *Alces alces*, cortisol, Diptera, flies, immune response, molt, moose, serum, sores

## Abstract

Moose (*Alces alces*) in boreal habitats feed and rest where they are exposed to Dipteran flies and the parasites they carry. We collected 31,905 flies during the summer from 12 habituated moose on the Kenai Peninsula, Alaska. Moose flies, *Haematobosca alcis* (Snow), Diptera: Muscidae—a species that completes its entire life cycle on or around moose—accounted for 91% of flies collected; the reminder of the flies collected included mosquitoes (Culicidae), black flies (Simuliidae), and deer flies (Tabanidae). Flies impose physiological costs for moose, e.g., vectors for parasites such as Legworm (*Onchocerca* spp.) which causes sores on the hind legs of moose. We found that the number of sores present on the hind legs of moose is positively correlated with body fat, which suggests a correlation between gains of energy and damage from flies. We also found that the number of sores is negatively correlated with serum albumin, which is indicative of an inflammatory response and body protein being used to repair injuries from flies and parasites. The number or type of flies present on a Moose were not correlated with the concentration of corticosteroids in saliva or feces. Flies do not elicit a stress response in moose even though the costs of repairing wounds and resisting infections of those wounds likely reduce gains of protein from summer foraging. Moose can tolerate the injuries from biting flies with regular gains from summer foraging but exposure to insect-borne parasites poses a risk to reproduction and survival.

## Teaser

Moose (*Alces alces*) in boreal habitats of Alaska are exposed to thousands of Dipteran flies throughout the summer months, with moose flies (*Haematobosca alcis*) making up the majority of fly residence on moose. Flies impose physiological costs such as tissue damage and loss of body protein, without eliciting a corticosteroid stress response in moose.

Wild ungulates are attacked by arthropods and arthropod-borne parasites while foraging to obtain enough energy and protein reserves for growth and reproduction (Samuel et al. 2001). Ectoparasites such as ticks or deer keds can contribute to alopecia which increases risk of winter mortality (Kynkäänniemi et al. 2014; Bondo et al. 2019) while vector-borne internal parasites such as filarioid nematodes contribute to skin lesions or other pathology (Grunenwald et al. 2016; Benedict et al. 2023a). The threat posed by nuisance hematophagous Diptera and other arthropods, and associated vector-borne pathogens, has considerable spatial and temporal variation and consequences for wild ungulate population health.

Ungulates use a combination of resistance and tolerance responses to contend with arthropods and their parasites (Rauw 2012; Hayward et al. 2014; Benedict and Barboza 2022). Ungulates can resist attack from arthropods by investing in behaviors (e.g., avoiding exposure to insects, grooming to displace arthropods) and morphologies (e.g., thick coats and skins) that reduce the number of bites and thus avoid ensuing infections. Behaviors that avoid arthropod attack incur a cost of movement as well as an opportunity cost (e.g., lost foraging time) with increased risks of mortality (e.g., predation or crossing water bodies, mountains, roads, and fences). Tolerance of repeated injury from arthropod attack may entail suppression of a stress response that is associated with costly behavioral and physiological reactions (e.g., corticosteroid response; Boonstra 2004; Defolie et al. 2020; Benedict et al. 2023b). Tolerance of arthropod bites incurs physiological costs for repair to wounded tissues as well as a cascade of immune responses (e.g., reactions to antigens from arthropod saliva, parasites, or microbes; Benedict and Barboza 2022; Benedict et al. 2023a). Costs of immune reactions vary over time from first exposure (Froy et al. 2019). Initial costs of immune response are high at first exposure to a pathogen, but subsequent exposures are less expensive when antibodies are maintained and reactivated by repeated exposure to the pathogen (Derting and Compton 2003). Sustained tolerance of a wide variety of pathogens is costly and may increase the risk of an autoimmune reaction (i.e., anti-self; Rauw 2012). The resulting suite of resistance and tolerance responses to insects depends upon the functional response of the host ungulate and the productivity of the habitat.

Moose (*Alces alces*) are attacked by many biting and nonbiting flies (Diptera) throughout the spring and summer months (Lankester and Samuel 2007; Benedict and Barboza 2022). Stretching from Alaska to Norway and south to Minnesota, flies from 14 families have been collected from Moose, including the Moose Fly, *Haematobosca alcis* (Snow). Diptera, Muscidae, which is thought to feed exclusively on moose and oviposit in fresh Moose feces (Lankester and Samuel 2007; Rolandsen et al. 2021; Benedict and Barboza 2022; Benedict et al. 2023b). The most evident costs of flies for Moose are the hind leg sores found in the area above the hock (the tibiotarsal joint) on adult Moose (Benedict et al. 2023a). While work is ongoing to definitively determine the cause of the sores, a recently discovered species of Legworm (*Onchocerca* sp.) has been linked to the formation of these sores in Moose (Benedict et al. 2023a). The vector of this species is yet to be determined, but related species of legworm in other ungulates are vectored by black flies (Diptera: Simuliidae; Benedict et al. 2023a). During June and July in Alaska, Moose populations molt, fly numbers increase, and *Onchocerca* parasites invade and create round sores on the hind legs of adult Moose (Benedict et al. 2023a). Up to 25 sores and associated inflammation have been observed on a single leg at one time on Moose in Alaska, with sores also being described in Michigan and Canada (Lankester and Samuel 2007; Benedict et al. 2023a). Even if impacts from these wounds remain local, tissue repair and immune response to local infection by parasites and secondary microbes may reduce summer mass gain of affected individuals (Samuel et al. 2001; Grunenwald et al. 2016; Benedict and Barboza 2022). Parasitic nematodes transferred by flies can cause life-threatening internal infections and neurological impairment (Grunenwald et al. 2016, 2018).

The wide variety of parasites associated with Moose reflects their resilience as a host species and strong functional response to seasonal pulses of food to ensure sufficient nutrition for survival and reproduction (Allen et al. 2017; Benedict et al. 2024). Growth varies with climate as annual cycles of summer supply and winter demand change across regions (Grøtan et al. 2009). Female Moose rely on high food intakes in a short summer of plant growth to grow their calves and to gain energy reserves for winter survival and the next pregnancy (Renecker and Hudson 1986; Grøtan et al. 2009; Shively et al. 2019). From 2 to 17 years old, female Moose produce 1 to 3 calves a year, having the greatest fecundity when primary plant production is high and winter severity is low (Boer 1992; Schwartz 1992, 2007; Schwartz and Hundertmark 1993; Sand 1996). Flies can reduce energy and nutrient intake of ungulates by reducing foraging time and displacing foraging from preferred areas (Hagemoen and Reimers 2002; Benedict and Barboza 2022; Benedict et al. 2024). Nematode parasites can also reduce body mass gains of ungulates by reducing protein intakes (Ezenwa 2004). The burden of biting flies and risk of parasitic infection are dynamic and influenced by global climate change. Consequently, changes in exposure to biting arthropods and associated parasites create the potential for Moose population regulation that could explain population declines observed in many regions of North America (Murray et al. 2006; Grunenwald et al. 2018).

We studied the tolerance of female Moose to fly exposure during summers in Alaska. We collected flies from adult Moose habituated to human contact as our measure of fly exposure. We measured the abundance and diversity of flies collected from the Moose in relation to environmental variables (vapor pressure deficit, ambient air temperature, wind, and habitat type) and time (time of day and Julian day) to understand the composition and phenology of the fly community on Moose. We measured changes in salivary and fecal corticosteroids to monitor the stress response to fly exposure through the summer as morphological resistance to flies declined through the annual molt. We predicted that increasing exposure to flies would not affect corticosteroid concentrations if Moose were tolerant of flies. Furthermore, we predicted that number of sores on the hind legs of Moose would be inversely related to body fat, showing the physiological costs of tolerance. We also predicted that serum protein concentrations would decline as leg sores increased and the costs of repairing wounds from fly parasites increased.

## Materials and methods

### Study system

This study was conducted at the Kenai Moose Research Center (MRC) operated by the Alaska Department of Fish and Game (ADFG) on the Kenai National Wildlife Refuge (60°N, 150°W) on the Kenai Peninsula, Alaska, United States. All procedures for care, handling, and experimentation of animals were approved by the Animal Care and Use Committee, ADFG, Division of Wildlife Conservation (IACUC protocol no. 0086) and by the Institutional Animal Care and Use Committee, Texas A&M AgriLife Research (AUP 2019-009A and 2021-009A), and followed ASM guidelines (Sikes et al. 2016). We studied free ranging tame adult (2 to 18 years old) female Moose (2015, *n* = 11; 2016, *n* = 12, 11 resampled from 2015; 2021, *n* = 12, 9 resampled from 2016) held in 2.6 km^2^ outdoor enclosures with seral stages of boreal forest, wetlands, open meadows, and lakes.

### Molt and sores

We observed the Moose for signs of molt and the appearance of sores at intervals of 5 to 20 days during 3 summers: 5 May to 13 July 2015; 3 May to 18 July 2016; and 19 May to 13 August 2021. Signs of molt and completion of molt were recorded at the time of observation, or were estimated from date-stamped photographs. Start of molt was characterized as loss of hair on the backs of the ears. Molt was considered complete when hair was missing or short across their entire body. Additionally, we paid careful attention to observing the loss of hair and emergence of sores on the area above the hock on the hind legs of the Moose. We also recorded the number of sores on each leg throughout the summer in 2021 (*n* = 12 moose).

### Body condition

We chemically immobilized the Moose as follows in 2021: 4 on 12 May, 2 on 20 May, 10 between 20 July and 22 July (when flies are abundant and sores are present; Benedict et al. 2023a), and 10 between 7 December and 8 December. Moose were immobilized with Thiafentanil oxalate (1 to 4 μg·kg^−1^ estimated body mass; 10 mg/mL; ZooPharm Wildlife Pharmaceuticals Inc., Windsor, Colorado) and Xylazine (30 to 50 μg·kg^−1^ estimated body mass; 100 mg·mL^−1^; Lloyd Laboratories, Shenandoah, Iowa). Immobilizing drugs were hand-injected deep into the shoulder muscle using a luer-lock syringe and 21 Ga × 25 mm hypodermic needle. Heart rate, respiration rate, and blood perfusion to the mucous membranes were monitored during immobilization. We collected up to 60 mL of blood via jugular venipuncture with a 20 Ga × 38 mm needle. Blood was sent to Zoetis Reference Laboratories (Mukilteo, Washington) for Complete Blood Count Equine and ClinChem25 panels. We also measured maximum rump fat thickness (MAXFAT) via ultrasonography (Ibex Pro, E.I. Medical Imaging, Loveland, Colorado) and converted it to ingesta-free body fat (IFBFAT) using the equation: IFBFAT = 5.61 + 2.05 × MAXFAT (Stephenson et al. 1998). We reversed immobilization of the animal within 45 min by injection of Atipamezole HCl (5 μg·kg^−1^ estimated body mass; ¼ dose intravenous, ¾ dose intramuscular; 5 mg·mL^−1^; Zoetis, Parsippany, New Jersey) and Naltrexone HCl (100 mg·mg^−1^ Thiafentanil oxalate intramuscular; 50 mg·mL^−1^; ZooPharm LLC, Laramie, Wyoming).

### Salivary and fecal cortisol

In order to develop our sampling techniques, we collected saliva from 1 Moose from 23 May to 19 August 10 times in 2019. We then collected saliva an average of 6 times and fecal samples an average of 8 times from 12 moose from 19 May to 13 August 2021. Moose were approached with a familiar food reward to ease salivary collection and elicit a salivary response. Prior to consuming the offered food, saliva was collected by swabbing between the teeth and gums using a synthetic swab (SalivaBio Children’s Swab; Salimetrics LLC, Carlsbad, California). We then placed each swab in a sterile tube (Swab Storage Tube; Salimetrics LLC, Carlsbad, California) which we kept on ice and later froze at −20 °C. Salivary cortisol was analyzed in duplicate using a cortisol ELISA assay by Salimetrics LLC (μg·dL^−1^; Salivary Cortisol; Carlsbad, California) and reported as the mean concentration in each sample (Millspaugh et al. 2002; Thompson et al. 2020a). We collected freshly deposited fecal samples (only when we knew which individual they were from) using a plastic bag, placed them on ice and later froze them at −20 °C. We dried the fecal samples to constant mass in a freeze-drier (Labconco Model 7752020, Kansas City, Missouri) and then milled them through a 1.0-mm mesh (Shively et al. 2019). Fecal samples were analyzed for glucocorticoids by radio-immunoassay by the Applied BioSciences Endocrinology Laboratory (µg·g^−1^; College Station, Texas; Thompson et al. 2020b), with assays validated by Crouse (2003). At the time of saliva and fecal collection, we measured ambient air temperature (°C) and relative humidity (hPa) using a portable weather meter (Kestrel 4400 Heat Stress tracker or Kestrel DROP, Kestrel, Boothwyn, Pennsylvania). Vapor pressure (vap_pres) was later calculated from ambient temperature and relative humidity by the equation: $\text{vap}\_\text{pres}=6.11\times 10^{\frac{7.5\;\text{Ta}}{\text{237.3+Ta}}}\;\times \;\frac{\text{relative }\;\text{ humidity}}{\text{100}}$ (Brice and Hall 2023).

### Flies

We collected flies from 1 Moose on 7 occasions in 2019 from 16 June to 19 August to establish the collection method. In 2021, we collected flies from 12 Moose on 5 to 8 occasions from 19 May to 13 August. Flies were collected by sweep netting near the skin surface with a 0.381-m diameter collapsible net (BioQuip, Rancho Dominquez, California) while a Moose was laying down conscious (Lloyd and Dipeolu 1974; Lankester and Samuel 2007; McGregor et al. 2019). Netting was focused on the hind end of the Moose, where most flies congregated. Netting stopped at 60 s or when the Moose stood up. Duration of net sweep was recorded along with habitat type and weather variables at the time of collection. We determined the habitat type by overlaying netting location, recorded by GPS (Oregon 650t; Garmin, Olathe, Kansas), with vegetation polygons (ArcMap 10.6.1; ESRI, Redland, California) of early-seral boreal forest (2 to 5 years postdisturbance, open canopy), mid-seral boreal forest (25 years postdisturbance), old growth boreal forest (65+ years postdisturbance), Black Spruce forest, wetland (kettle ponds and/or sphagnum peat bogs with areas of standing water), and open meadow (Thompson et al. 2021). We measured ambient air temperature (°C), wind speed (m·s^−1^), and relative humidity (hPa) with Kestrel 4400 Heat Stress tracker or Kestrel DROP and Kestrel 1000 Pocket Wind Meter (Kestrel, Boothwyn, Pennsylvania). Flies were killed by acetone exposure and stored frozen for analysis. Insect samples were transported under a USDA Veterinary Permit (139420 Research) to our laboratory at Texas A&M University (College Station, Texas). We identified the flies morphologically and counted them under a dissection microscope into the following groups: mosquitoes (Culicidae), moose flies (Muscidae), coprophagous flies (various families), black flies (Simuliidae), horse and deer flies (Tabanidae), snipe flies (Rhagionidae), and other flies (Benedict et al. 2023b).

### Calculations and statistics

We performed all statistical analysis in STATA version 16.0 (StataCorp 2019). We used a reverse stepwise selection procedure for all models, which removed coefficients that were not significantly different from 0. We used the robust “sandwich estimator” for standard errors to relax assumptions of normal distribution and homogeneity of variances (Rabe-Hesketh and Skrondal 2010). All statistical significance was set at *P* ≤ 0.05.

We used robust regression to examine the effects of the average number of hind leg sores (max_sores) on IFBFAT, blood proteins (total protein [protein], albumin, globulins, fibrinogen), and blood cells (eosinophils and lymphocytes) in July: max_sores = IFBFAT + protein + albumin + globulins + fibrinogen + eosinophils + lymphocytes + ɛ.

We used mixed-effects regression with individual Moose as random effects to account for repeated measures of dependent variables to examine the effects of ambient air temperature (Ta), time of day (time), and Julian day on salivary cortisol levels (salivary cortisol): salivary cortisol = Ta + time + julian + ɛ. The same methods were repeated for fecal corticosteroids (fecal cortisol): fecal cortisol = Ta + time + julian + ɛ.

To examine the effects of environmental variables on total fly abundance netted per second (flies; not grouped by taxa) we regressed vapor pressure, ambient air temperature, Julian day, wind, time of day, habitat type (habitat), and individual Moose (individual) against flies netted per second: flies = vap_pres + Ta + julian + wind + time + habitat + individual + ɛ. The same methods were repeated for each of the fly groups (mosquitoes, moose flies, coprophagous flies, black flies, horse and deer flies, snipe flies, other flies). The final significant model for flies netted per second (of all groups combined) was then used to predict fly numbers at every saliva and fecal collection by predicting the margins using each corresponding individual and Julian day. Prediction accuracy was checked by regressing observed against predicted flies netted per second. We then used mixed-effects regression with individual Moose and Julian day as random effects to account for repeated measures of dependent variables to examine the effects of predicted flies (predict_flies) on salivary cortisol levels: salivary cortisol = predict_flies + ɛ. The same method was used for fecal corticosteroids: fecal cortisol = predict_flies + ɛ. The same method was repeated again for fecal corticosteroids but using predicted flies from the prior Julian day to account for a 24-h lag in fecal glucocorticoids in response to a stressor (Crouse 2003; Lechner et al. 2010).

## Results

**Fig. 1.**
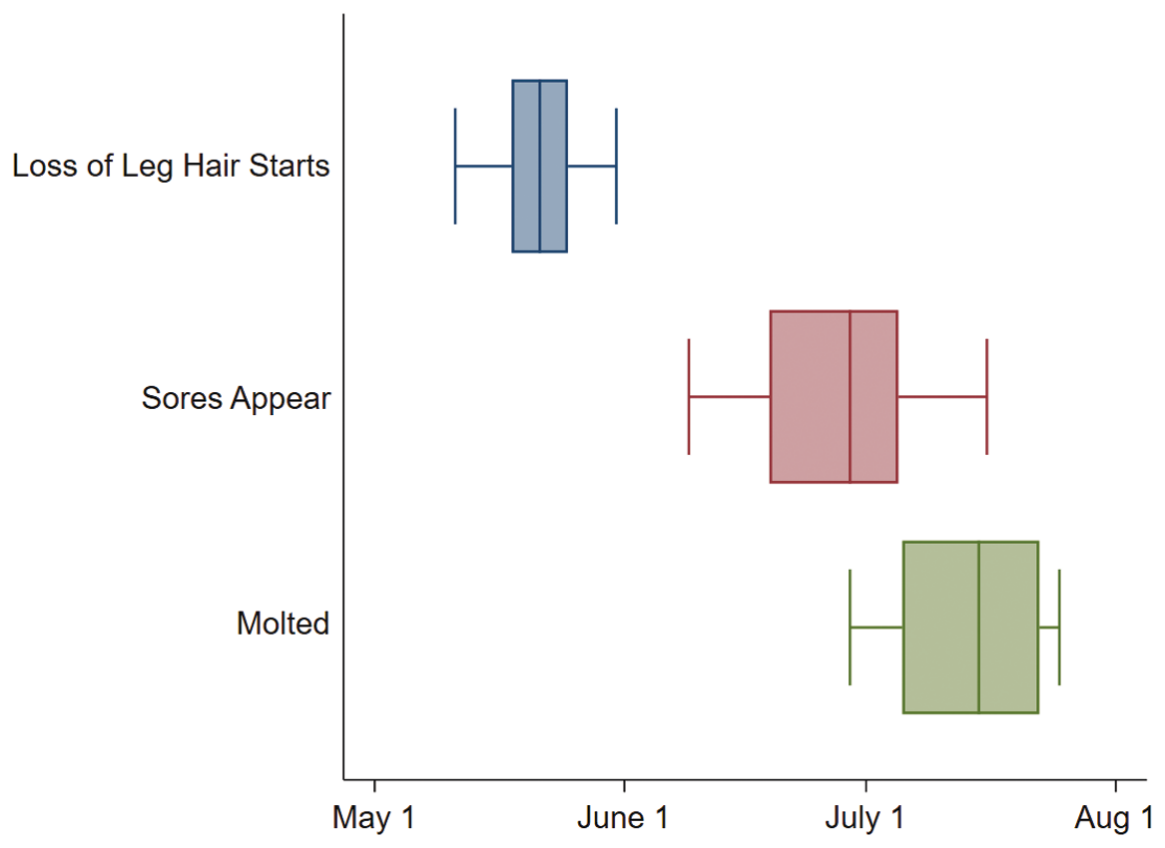
Box plots with median, 25th percentile, 75th percentile, and range (bar) of the start of loss of hair on the area above the hock (*n* = 32; left box plot), emergence of sores in this area (*n* = 33; middle box plot), and completion of molt (*n* = 28; right box plot) in female adult Moose at the Kenai Moose Research Center, Kenai Peninsula, Alaska, United States.

Observations for start and stop of whole-body molt, loss of hair from above the hock, and presence of sores were made across 15 female Moose (2015 *n* = 11; 2016 *n* = 12; 2021 *n* = 12). Signs of whole-body molt starting were seen on the first day of each study year (5 May 2015; 3 May 2016; and 19 May 2021). Moose had completed molting 14 July ± 8 days (Fig. 1). Loss of hair from above the hock began 21 May ± 5 days, and was followed by the appearance of sores on 28 June ± 11 days (Fig. 1). All Moose had sores by 5 July and continued to have sores through August (Fig. 1); sores were counted in July.

**Fig. 2.**
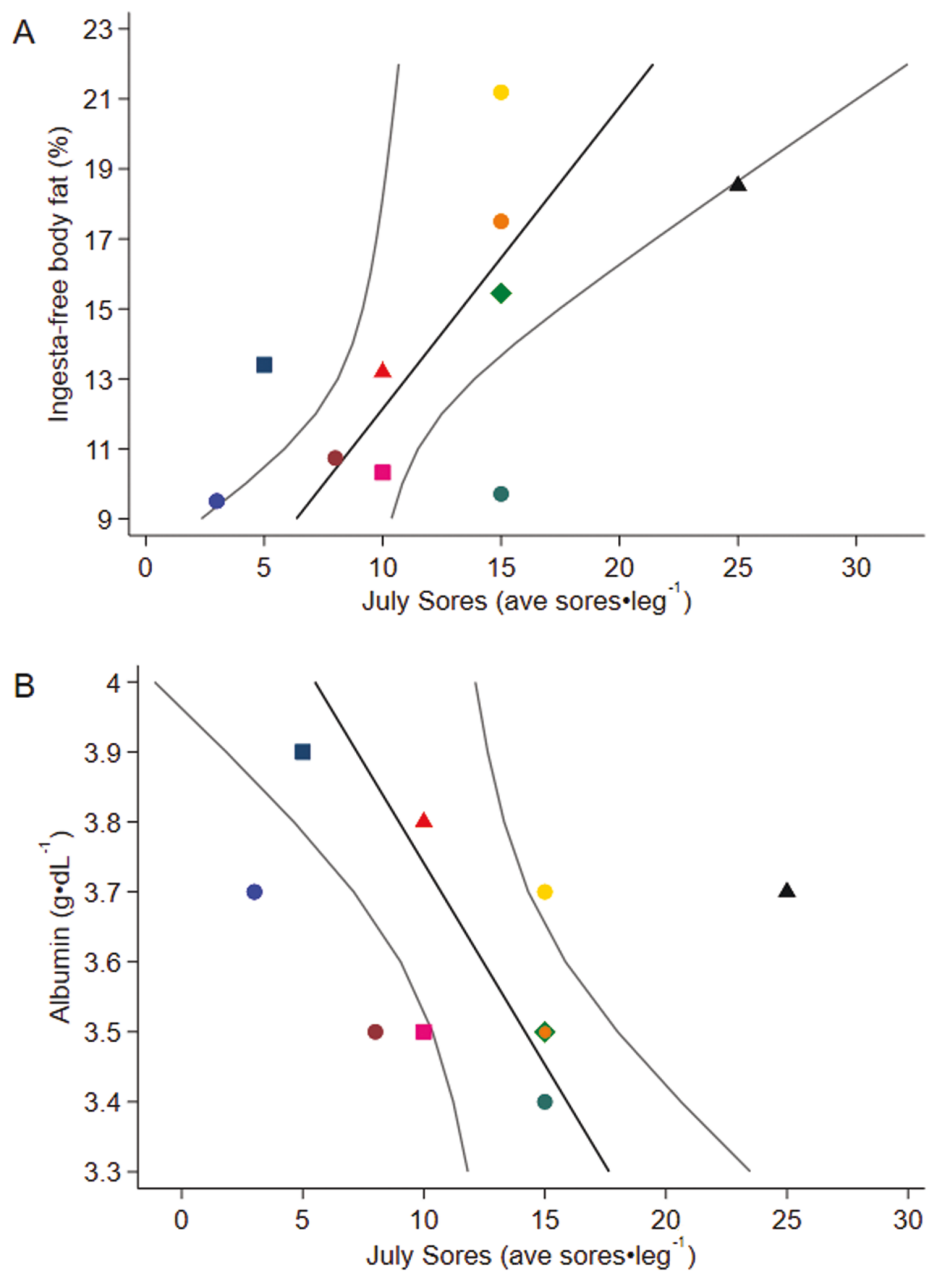
Marginal predictions of ingesta-free body fat (%; A) and serum albumin (g·dL^−1^; B) on the average number of sores per hind leg of female adult Moose (*n* = 10 series of colors, individuals) in July at the Kenai Moose Research Center, Kenai Peninsula, Alaska, United States, based on robust linear regression (*R*^2^ = 0.599, *P* = 0.014).

IFBFAT increased from May to July but had declined by December (Supplementary Data SD1). In July, the number of sores present (3 to 25 sores) on the hind legs of Moose was positively correlated with the IFBFAT of a Moose (Fig. 2A), while the number of sores was negatively correlated with serum albumin (Fig. 2B; *n* = 10; *R*^2^(1) = 0.60, *P* = 0.014; Fig. 2 and Supplementary Data SD2). The number of sores was not significantly related to serum protein, serum globulin, serum fibrinogen, or to the counts of eosinophils and lymphocytes in whole blood.

Saliva was collected from 11 Moose for a total of 73 collections, with 2 collections removed from analysis for extreme values and 1 for contamination with food. Salivary cortisol ranged from 0.02 to 0.12 μg·dL^−1^ but was not significantly related to ambient air temperature, time of day, or Julian day. Fecal samples were collected from 12 Moose for a total of 95 collections. Fecal corticosteroids declined significantly with the time of day from 3.17 µg·g^−1^ at 9:00 AM to 2.90 µg·g^−1^ at 6:00 PM (χ^2^(1) = 5.12, *P* = 0.024; Supplementary Data SD3), but ambient air temperature and Julian day were not significantly related to fecal corticosteroids.

**Fig. 3.**
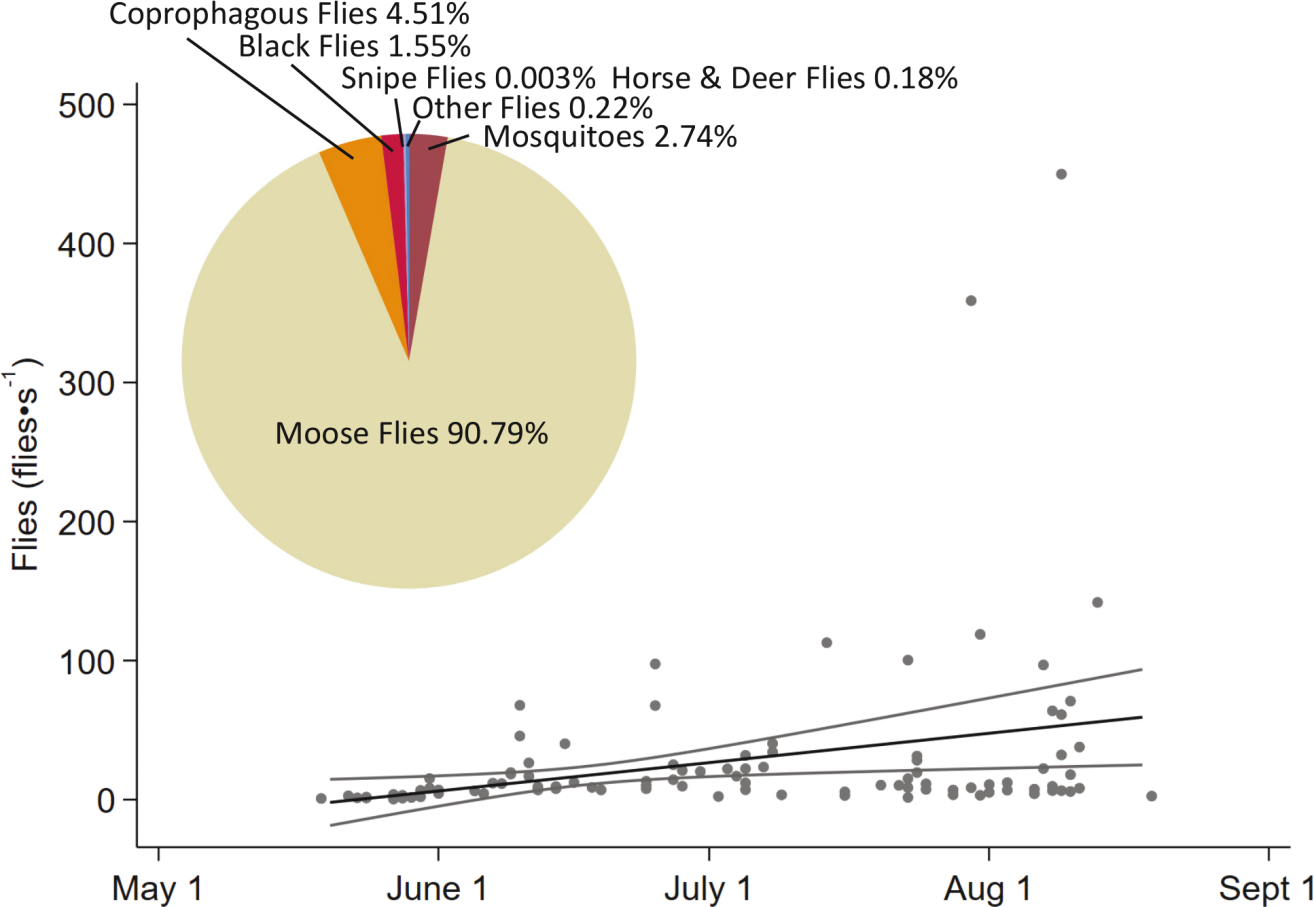
Marginal predictions and observations (dots), of Julian day on flies (flies·s^−1^) netted from female adult Moose (*n* = 12) at the Kenai Moose Research Center, Kenai Peninsula, Alaska, United States, based on linear regression (*R*^2^ = 0.216, *P* = 0.007). Pie chart shows the percent of total flies collected in each group (colors).

**Fig. 4.**
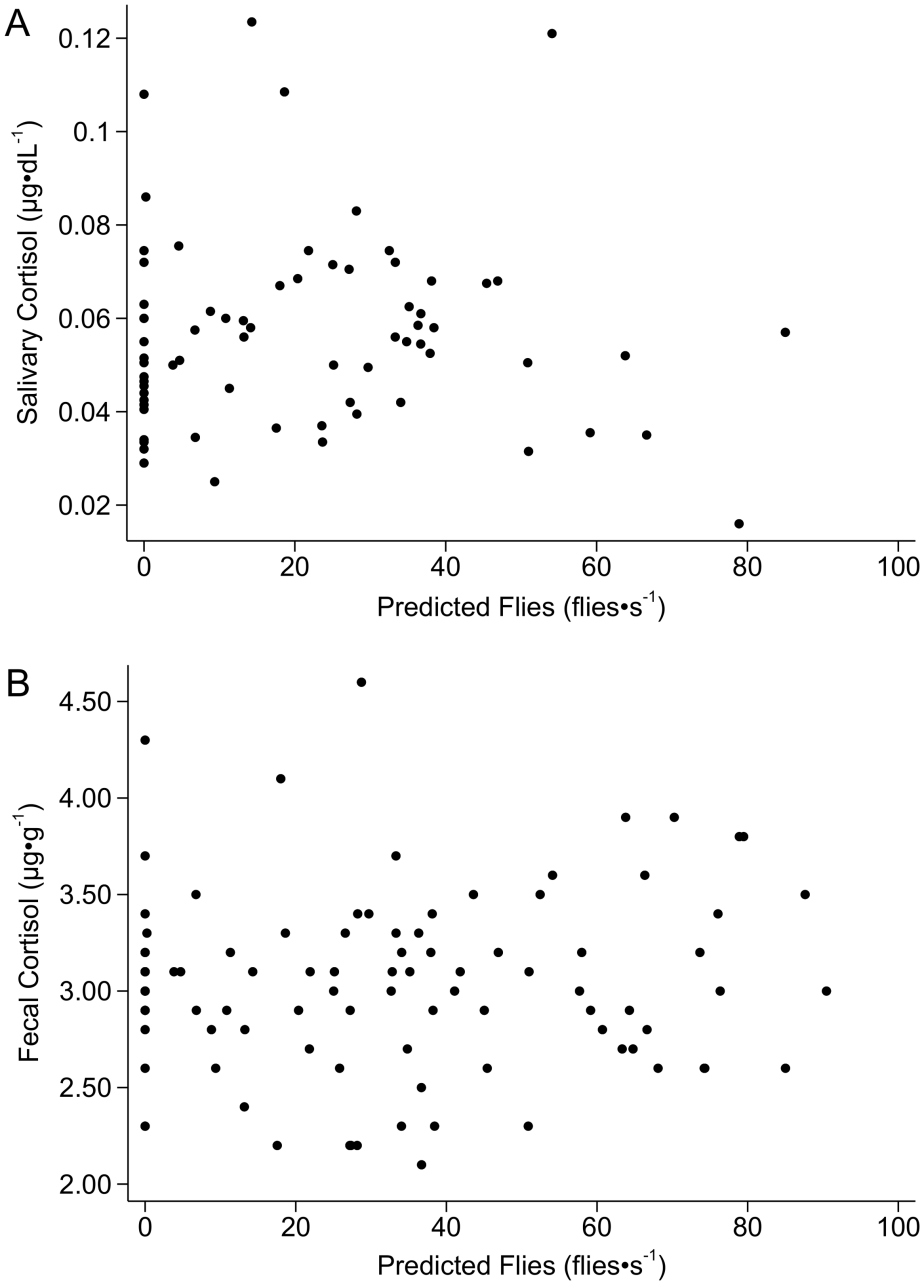
Flies (flies·s^−1^) predicted to occur at time of collection and the stress response of female adult Moose (*n* = 12) at the Kenai Moose Research Center, Kenai Peninsula, Alaska, United States, measured as cortisol in saliva (A; µg·dL^−1^) or corticosteroids in feces (B; µg·g^−1^).

Flies were collected from 12 Moose for a total of 98 collections. A total of 31,905 flies were collected—most of which were moose flies (28,968, 90.79%; Fig. 3)—the remainder were coprophagous or necrophagous flies (1,440, 4.51%), mosquitoes (873, 2.74%), black flies (494, 1.55%), horse and deer flies (58, 0.18%), other flies (71, 0.22%), and snipe flies (1, <0.01%). Up to 450 flies were netted from a single Moose in 1 s (Fig. 3). Julian day and individual Moose were the only significant effects on the number of flies (*R*^2^ = 0.216, *P* = 0.007; Fig. 3; Supplementary Data SD4 and SD5) and the number of moose flies (*R*^2^ = 0.206, *P* = 0.007; Supplementary Data SD4 and SD5) netted per second. It should be noted that the proportion of explained variance is low, however, when the 2 extreme values for flies are dropped the variance improved (flies *R*^2^ = 0.439, *P* = 0.002; moose flies *R*^2^ = 0.431, *P* < 0.001). The number of mosquitoes netted per second were significantly affected by Julian day, ambient air temperature, wind, time of day, and vapor pressure (*R*^2^ = 0.203, *P* = 0.000; Supplementary Data SD4 and SD5). We used the model of all flies netted per second (all groups combined) to estimate the number of flies experienced by each Moose at every sampling event for saliva and feces. Predicted and observed numbers of flies were linearly related at a slope close to 1 (1.139 ± 0.211; Supplementary Data SD5). Neither salivary cortisol nor fecal corticosteroids were significantly related to the predicted number of flies on the day of collection of saliva (χ^2^(1) = 0.01, *P* = 0.92; Fig. 4A) or feces (χ^2^(1) = 0.45, *P* = 0.50; Fig. 4B), or 24 h prior to collection of feces (χ^2^(1) = 0.45, *P* = 0.50).

## Discussion

Moose molt in summer to reduce insulation as air temperatures increase, which consequentially leaves them without a layer of hair to act as a barrier to fly attacks. As adult female Moose completed molt in July (Fig. 1), flies were abundant (Fig. 3) and particularly concentrated on the area above their hocks, blanketing this area, contributing to the formation of sores in late summer (Fig. 1). These sores are likely created by legworm transmitted by black flies (Benedict et al. 2023a), but this has yet to be confirmed. In comparison, Moose calves are born in the spring with a fuzzy natal coat and molt to a winter coat, never directly exposing the skin on their legs (unless injured) to flies, and thus never forming sores (Benedict et al. 2023a).

The relationship between fly exposure and body fat was unexpected. Summer body fat, measured as IFBFAT, was positively related to the number of hind leg sores (Fig. 2A). Moose with more body fat might have more surface area on their legs for sores to form and flies to concentrate, or the flies may be attracted to the increased lipid content, implying that foraging indirectly leads to more damage from flies. It should be noted that this relationship is only indirect; a recent study found that the act of foraging does not expose Moose to more flies (Benedict et al. 2024). Moose spent most of their time in early-seral boreal forests where forage is the most abundant and flies are sparse, resting in the most fly-abundant Black Spruce forests when the need for cooling and resting takes priority (Benedict et al. 2024). Further data are needed to draw definitive conclusions about the cause of the relationship between body fat and hind leg sores, including relationships between net change in body fat across the summer.

We saw a negative relationship between serum albumin concentration and the number of sores on a Moose (Fig. 2B). A decrease in albumin is generally indicative of disease, malnutrition, and blood loss (Kaneko 1997; Crouse 2003; Reshetnyak et al. 2021), and in our study indicative of inflammatory response and body protein being used to repair sores (Franzmann and Leresche 1978). Our albumin measurement (3.4 to 3.9 g⋅dL^−1^) was on the lower end of the range of past adult female MRC Moose (3.8 to 5.2 g⋅dL^−1^) in the month of July (Franzmann and Bailey 1977), and below the threshold of 4.5 g⋅dL^−1^ for moose in average or better condition (Franzmann and Leresche 1978). Past studies found a negative relationship between albumin and body condition in Moose (Franzmann Index condition classes based on physical status and rump fat; Franzmann and Bailey 1977). Albumin is plastic, responding rapidly to changes in nutrition and inflammation, explaining variation in survival of Soay Sheep (*Ovis aries*) independent of body weight and immunoglobulins (Garnier et al. 2017). The low serum albumin values in our data are consistent with an elevated demand for protein to repair sores and damage from flies.

Moose are tolerant of flies throughout the summer; up to 1,515 flies (the majority of which were moose flies) were netted from a single Moose in 60 s, with no measurable response in salivary cortisol (Fig. 4). Salivary cortisol reflects cortisol in the blood and peaks 20 to 30 min after the onset of stress, while fecal glucocorticoids reflect stressors within 15 to 22 h (Crouse 2003; Merlot et al. 2011; Sheriff et al. 2011; Majchrzak et al. 2015). Previous studies of Moose have looked at body condition and stress in relationship to environmental variables (Becker et al. 2010; Thompson et al. 2020a), but the effect of flies on the stress response of adult Moose has not been characterized. The only significant effect on salivary or fecal corticosteroids that we found was time of day, which is consistent with diel rhythms in the excretion of glucocorticoids in feces (Jachowski et al. 2015). Our measurements of salivary cortisol (0.02 to 0.12 µg⋅dL^−1^; Fig. 4A) were on the lower end of what has been observed for adult female Moose (~0 to 3.0 μg·dL^−1^; Thompson et al. 2020a) and Moose calves (0 to 0.2 μg·dL^−1^; Benedict et al. 2023b) at the MRC (without any added stresses) using the same assay, but still within the sensitivity of the assay (0.012 μg·dL^−1^ detection sensitivity; Salimetrics LLC, Carlsbad, California). Our measurement of fecal glucocorticoids (2.1 to 4.6 µg·g^−1^; Fig. 4B) overlapped with ranges found in past studies of adult female MRC Moose (~1.0 to 2.75 µg·g^−1^, without any added stresses; Thompson et al. 2020a) but were on the higher end, possibly due to between-year weather differences. Thompson et al. (2020a, 2020b) found a correlation between increases in salivary cortisol and rapid increases in ambient air temperature with chemical immobilization and capture, indicating that salivary cortisol can be used to measure stress in Moose similar to serum, hair, and feces (Bubenik et al. 1994; Crouse 2003; Madslien et al. 2020; Spong et al. 2020; Rosenblatt et al. 2021). While Moose may not be releasing glucocorticoid hormones as an emergency response to flies, we did observe Moose displaying anti-fly behaviors by shaking their head, running, blowing their nose, trying to nudge flies off their hind legs (most cannot reach the back of their legs and none can reach their rump), and twitching in response to flies. This suggests that they still may be experiencing stress without a release of glucocorticoid hormones, showing their tolerance of flies. Moose calves that must follow their mothers and incur the same exposure to flies also do not increase salivary cortisol as the number of flies increase (Benedict et al. 2023b). Exposure to flies from birth is not a new phenomenon for boreal Moose in Alaska, causing habituation and physiological tolerance, which is contrary to the experience of moose in northeastern United States in response to Winter Tick (*Dermacentor albipictus*; Rosenblatt et al. 2021). Winter ticks have been on the rise in the northeast for the past 20 years. The lack of tolerance, behavioral, or physiological adaptations of Moose in that region to ticks has been seen in the form of calf stress hormone metabolite concentrations rising in relation to Winter Tick infestations during food-stressed winter months (Rosenblatt et al. 2021).

The majority of flies collected directly from adult Moose (90.79%; Fig. 3) and calves (68.4%; Benedict et al. 2023b) are moose flies, with abundances increasing through summer, but less than 0.03% of flies collected off of a host using CO_2_-baited light traps and sticky traps were moose flies (Benedict et al. 2024). The abundance of flies in Moose habitat is significantly affected by environmental variables such as vapor pressure, ambient air temperature, and habitat type (Benedict et al. 2024)—while the abundance of flies on a Moose is not. Instead, there were significant differences between the number of flies netted and the individual Moose (Supplementary Data SD5). These comparisons suggest that Moose are their own moving habitats for flies, and in particular for moose flies, in addition to experiencing other species of flies in the habitat that they use. The costs and benefits of the insect–host relationship between moose flies and Moose awaits further studies of the transfer of nutrients from Moose to fly and of parasites from fly to Moose. Higher resolution taxonomic identification, sex ratios, reproductive status, and parasitic status of flies should be included in future studies, along with further microbial analyses to further parse out Moose–fly relationships.

Moose are able to tolerate flies because they attain high intakes of energy and protein that can offset the added costs of injury and tissue repair (Shively et al. 2019). Tolerance of parasites carried by flies may vary widely because exposure to nematode parasites depends on transmission dynamics of vector and host populations (Hayward et al. 2014; Laaksonen et al. 2015; Buckingham and Ashby 2022).

## Supplementary data

Supplementary data are available at *Journal of Mammalogy* online.

**Supplementary Data SD1.** Ingesta-free body fat (%) measurements across female adult Moose (*n* = 12 series of colors, individuals) at the Kenai Moose Research Center, Kenai Peninsula, Alaska, United States, across Julian days.

**Supplementary Data SD2.** Results for the robust regression of ingesta-free body fat (IFBFAT), blood proteins (total protein, albumin, globulins, fibrinogen), and blood cells (eosinophils and lymphocytes) on the average number of hind leg sores observed on a Moose, in July at the Kenai Moose Research Center, Kenai Peninsula, Alaska, United States. Standardized beta coefficients only of significant fixed effects (*P* < 0.05) are shown.

**Supplementary Data SD3.** Results for the regression of the effects of ambient air temperature (Ta), time of day (time), and Julian day on fecal corticosteroids (fecal cortisol) of Moose at the Kenai Moose Research Center, Kenai Peninsula, Alaska, United States. Individual Moose were included as random effects to account for repeated measures of dependent variables. Standardized beta coefficients only of significant fixed effects (*P* < 0.05) are shown.

**Supplementary Data SD4.** Marginal predictions of Julian day on flies (*R*^2^ = 0.216, *P* = 0.007), moose flies (*R*^2^ = 0.206, *P* = 0.007), mosquitoes (*R*^2^ = 0.203, *P* = 0.000), and other flies (*R*^2^ = 0.041, *P* = 0.043) netted per second from female adult Moose (*n* = 12) at the Kenai Moose Research Center, Kenai Peninsula, Alaska, United States, based on linear regression.

**Supplementary Data S5.** Results for the regression of the effects of vapor pressure (vap_pres), ambient air temperature (Ta), Julian day (julian), wind, time of day (time), habitat type (habitat), and individual Moose (individual) against flies netted per second (combined [flies] and by group) at the Kenai Moose Research Center, Kenai Peninsula, Alaska, United States. Standardized beta coefficients only of significant fixed effects (*P* < 0.05) are shown.

gyae081_suppl_Supplementary_Data_SD1

gyae081_suppl_Supplementary_Data_SD2

gyae081_suppl_Supplementary_Data_SD3

gyae081_suppl_Supplementary_Data_SD4

gyae081_suppl_Supplementary_Data_SD5

## Data Availability

Data available upon request, subject to data sharing agreement.
