# Supplementary material for: Wounded but unstressed: Moose tolerate injurious flies in the boreal forest
Source: J Mammal. 2024 Aug 7;105(5):1166–74. doi: 10.1093/jmammal/gyae081 (PMC11520747; doi:10.1093/jmammal/gyae081)
Supplement: gyae081_suppl_Supplementary_Data_SD1 [file gyae081_suppl_supplementary_data_sd1.docx]

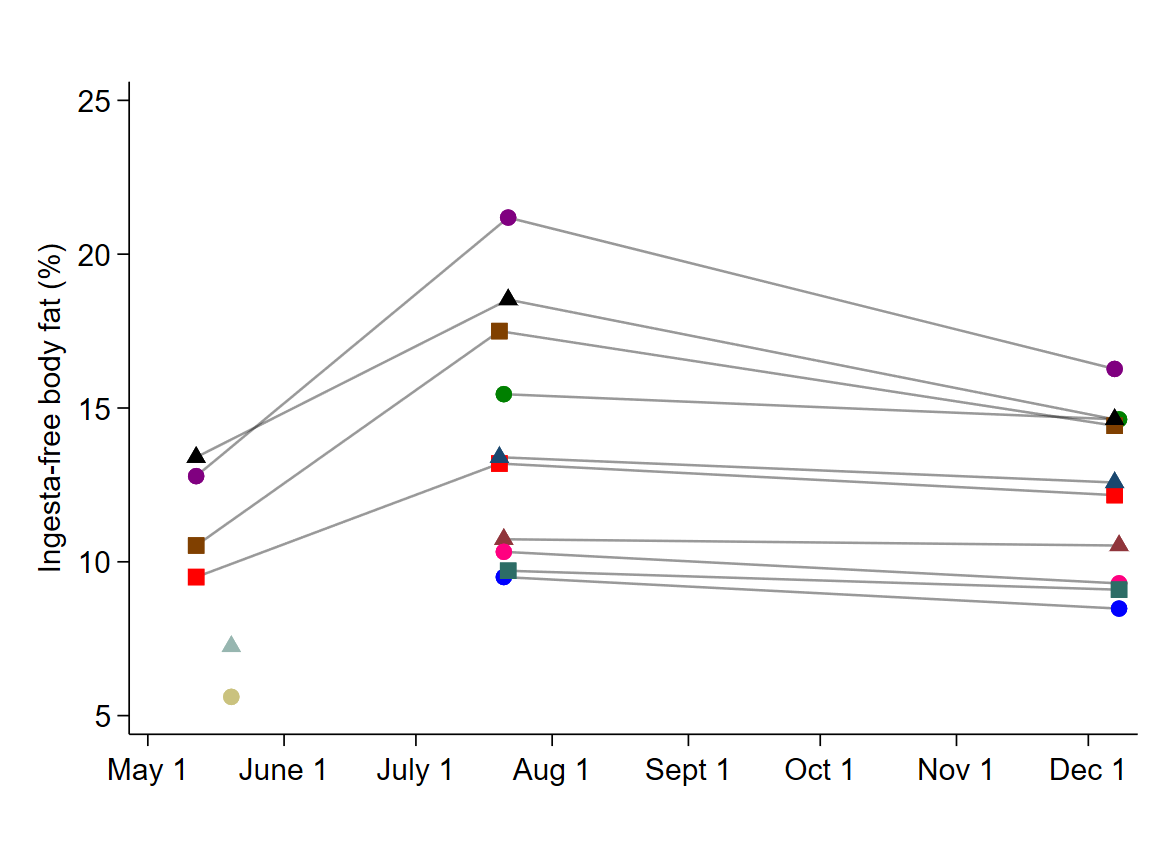


Supplementary Data S1.—Ingesta-free body fat (%) measurements across female adult moose (n=12 series of colors, individuals) at the Kenai Moose Research Center, Kenai Peninsula, Alaska, USA, across Julian days.
