# Supplementary material for: Wounded but unstressed: Moose tolerate injurious flies in the boreal forest
Source: J Mammal. 2024 Aug 7;105(5):1166–74. doi: 10.1093/jmammal/gyae081 (PMC11520747; doi:10.1093/jmammal/gyae081)
Supplement: gyae081_suppl_Supplementary_Data_SD2 [file gyae081_suppl_supplementary_data_sd2.docx]

Supplementary Data S2.—Results for the robust regression of ingesta-free body fat (IFBFAT), blood proteins (total protein, albumin, globulins, fibrinogen) and blood cells (eosinophils and lymphocytes) on the average number of hind leg sores observed on a moose, in July at the Kenai Moose Research Center, Kenai Peninsula, Alaska, USA. Standardized beta coefficients only of significant fixed effects (*P*<0.05) are shown.

|  | Dependent Variable (Y) | |
| --- | --- | --- |
| Parameters and main effects | | Hind Leg Sores |
| Observations | | 10 |
| R^2^ | | 0.60 |
| Intercept | | 58.78 |
| IFBFAT | | 1.16 |
| Albumin | | -17.36 |
|  | |  |
|  | |  |
|  | |  |
