# Supplementary material for: Wounded but unstressed: Moose tolerate injurious flies in the boreal forest
Source: J Mammal. 2024 Aug 7;105(5):1166–74. doi: 10.1093/jmammal/gyae081 (PMC11520747; doi:10.1093/jmammal/gyae081)
Supplement: gyae081_suppl_Supplementary_Data_SD3 [file gyae081_suppl_supplementary_data_sd3.docx]

Supplementary Data S3.—Results for the regression of the effects of ambient air temperature (Ta), time of day (time), and Julian day on fecal corticosteroids (fecal cortisol) of moose at the Kenai Moose Research Center, Kenai Peninsula, Alaska, USA. Individual moose were included as random effects to account for repeated measures of dependent variables. Standardized beta coefficients only of significant fixed effects (*P*<0.05) are shown.

|  | Dependent Variable (Y) |
| --- | --- |
| Parameters and main effects | Fecal Cortisol |
| Observations | 95 |
| *X*^2^ [df] | 5.12 [1] |
| Intercept | 3.51 |
| Time | -0.91 |
