# Supplementary material for: Wounded but unstressed: Moose tolerate injurious flies in the boreal forest
Source: J Mammal. 2024 Aug 7;105(5):1166–74. doi: 10.1093/jmammal/gyae081 (PMC11520747; doi:10.1093/jmammal/gyae081)
Supplement: gyae081_suppl_Supplementary_Data_SD4 [file gyae081_suppl_supplementary_data_sd4.docx]

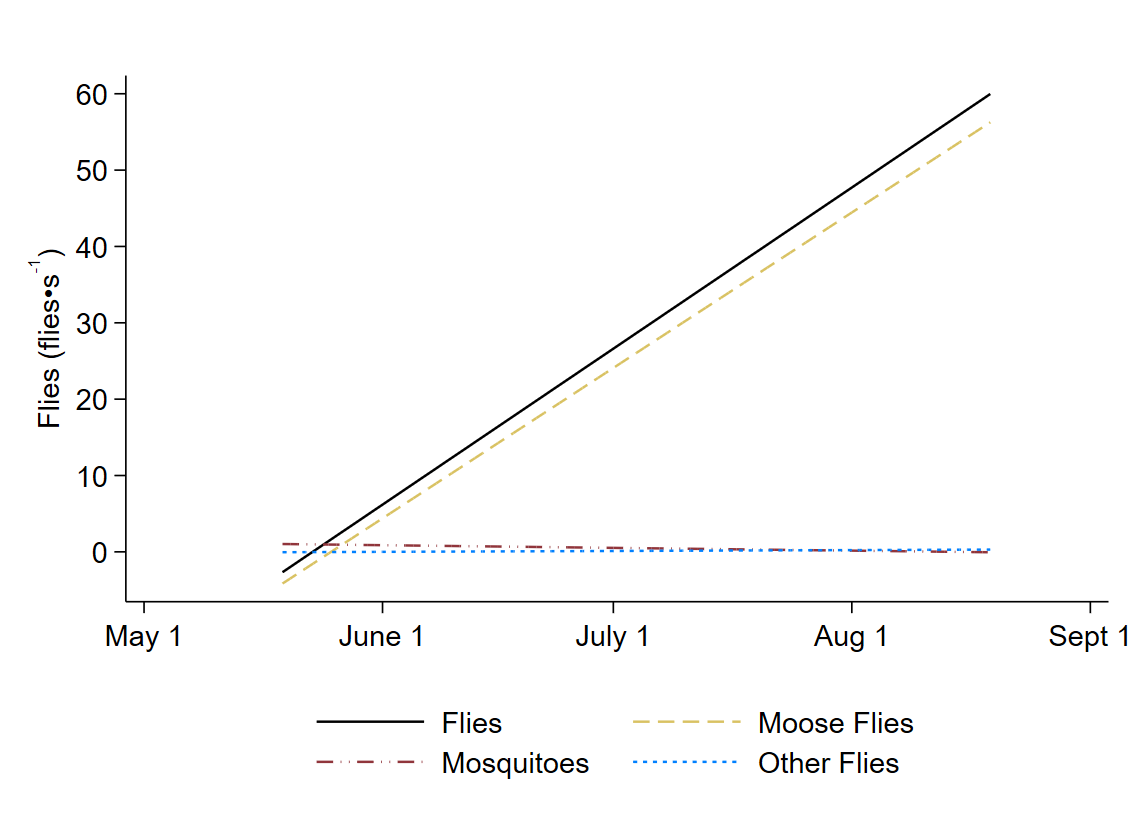
 Supplementary Data S4.—Marginal predictions of Julian day on flies (R^2^ = 0.216, *P* = 0.007), moose flies (R^2^ = 0.206, *P* = 0.007), mosquitoes (R^2^ = 0.203, *P* = 0.000), and other flies (R^2^ = 0.041, *P* = 0.043) netted per second from female adult moose (n=12) at the Kenai Moose Research Center, Kenai Peninsula, Alaska, USA, based on linear regression.
