# Supplementary material for: Wounded but unstressed: Moose tolerate injurious flies in the boreal forest
Source: J Mammal. 2024 Aug 7;105(5):1166–74. doi: 10.1093/jmammal/gyae081 (PMC11520747; doi:10.1093/jmammal/gyae081)
Supplement: gyae081_suppl_Supplementary_Data_SD5 [file gyae081_suppl_supplementary_data_sd5.docx]

Supplementary Data S5.—Results for the regression of the effects of vapor pressure (vap_pres), ambient air temperature (Ta), Julian day (julian), wind, time of day (time), habitat type (habitat), and individual moose (individual) against flies netted per second (combined (flies) and by group) at the Kenai Moose Research Center, Kenai Peninsula, Alaska, USA. Standardized beta coefficients only of significant fixed effects (*P*<0.05) are shown.

|  | Dependent Variable (Y) | | | | | |  |
| --- | --- | --- | --- | --- | --- | --- | --- |
| Parameters and main effects | Flies | Moose Flies | Coprophagous Flies | Mosquitoes | Black Flies | Horse and Deer Flies | Other Flies |
| Observations | 98 | 98 | 98 | 98 | 98 | 98 | 98 |
| R^2^ | 0.22 | 0.21 | 0.02 | 0.20 | 0.04 | 0.02 | 0.04 |
| Intercept | -115.31 | -111.70 | -1.45 | -0.64 | -1.52 | -0.07 | -0.57 |
| Julian | 0.68 | 0.66 | ̶ | -0.01 | ̶ | ̶ | 0.00 |
| Ta | ̶ | ̶ | 0.17 | ̶ | ̶ | 0.01 | ̶ |
| Ta^2^ | ̶ | ̶ | ̶ | -0.01 | -0.01 | ̶ | ̶ |
| Wind^2^ | ̶ | ̶ | ̶ | -0.48 | ̶ | ̶ | ̶ |
| Time | ̶ | ̶ | ̶ | -1.78 | ̶ | ̶ | ̶ |
| Vap_Pres | ̶ | ̶ | ̶ | 0.09 | ̶ | ̶ | ̶ |
| Individuals |  |  | ̶ | ̶ | ̶ | ̶ | ̶ |
| Individual 1 | 40.52 | 32.14 |  |  |  |  |  |
| Individual 2 | 39.08 | 33.62 |  |  |  |  |  |
| Individual 3 | 35.79 | 35.26 |  |  |  |  |  |
| Individual 4 | 5.26 | 4.29 |  |  |  |  |  |
| Individual 5 | 51.12 | 48.82 |  |  |  |  |  |
| Individual 6 | -9.44 | -9.04 |  |  |  |  |  |
| Individual 7 | 30.52 | 26.61 |  |  |  |  |  |
| Individual 8 | 0.20 | 0.10 |  |  |  |  |  |
| Individual 9 | 55.32 | 53.58 |  |  |  |  |  |
| Individual 10 | -1.15 | -1.2 |  |  |  |  |  |
| Individual 11 | 2.97 | 2.76 |  |  |  |  |  |

Regression of predicted versus observed flies (flies•s^-1^) plotted (blue line and circles) in comparison to a 1:1 comparison (orange line) (R^2^ = 0.233, *P* < 0.001).
